# Holding CoVID in check through JAK? The MPN-approved compound ruxolitinib as a potential strategy to treat SARS-CoV-2 induced systemic hyperinflammation

**DOI:** 10.1038/s41375-020-0898-6

**Published:** 2020-06-11

**Authors:** F. Heidel, A. Hochhaus

**Affiliations:** Klinik für Innere Medizin II, Universitätsklinikum, Jena, Germany

**Keywords:** Infectious diseases, Molecularly targeted therapy

Shortly after the onset of the CoVID-19 pandemic, the view on the disease in the medical community was dominated by the high contagiosity of SARS-CoV-2 and a rather mild disease phenotype that most patients would experience. Mainly older patients with significant comorbidities seemed to be at risk for a severe disease phenotype as indicated by high mortality rates in the elderly population. This view has somehow changed, as also younger patients may develop severe symptoms requiring hospitalization and even children may experience serious consequences of CoVID-19 infections, such as Kawasaki-like syndromes [1]. Patients suffer from a systemic (hyper-)inflammatory response syndrome that eventually results in severe pneumonia, acute respiratory distress syndrome (ARDS) or inflammation of the vascular system and is frequently the cause of death. The SARS-CoV-2 induced immune response resembles other systemic inflammatory response syndromes characterized by activation of CD4 and CD8 positive T cells, NK cells and monocytes and secretion of the related cyto- and chemokines. Recent reports investigating serum from CoVID-19 patients indicated elevated levels of IL6, IL9, IL13, GM-CSF, IFNγ, IL1b, IL8, and IL17 compared with healthy controls and even more pronounced IL2, IL7, IL10, TNFα and G-CSF in those requiring intensive care [2]. This report was confirmed by other groups describing elevated serum levels of IL10, IL6, TNFα and also enhanced TNFα target gene expression [3].

Janus-kinase (JAK) molecules have been linked to inflammatory processes since they serve as signaling nodes downstream of major cytokine receptors [4]. Therefore, most inflammatory conditions involve JAK signal transduction and are dependent on JAK-activity. The JAK-family consists of four members (JAK1, JAK2, JAK3, and TYK2) that have distinct association patterns to receptors and differentially activate downstream effectors such as *signal transducer and activator of* transcription (STATs), *mitogen-activated protein kinases* (MAPK), and *protein kinase B pathway* (PI3K/AKT) [5]. Accordingly, JAKs can be pathologically activated by either excessive ligand binding (e.g., under inflammatory conditions) or by activating mutations that promote differentiation and cytokine production in immune- and hematopoietic progenitor cells.

Development of JAK inhibitors has revolutionized the field of several inflammatory diseases such as myeloproliferative neoplasms (MPN), graft-versus-host disease (GvHD), hemophagocytic lymphohistiocytosis (HLH) among others. In MPN patients pharmacologic JAK1/2-inhibitor treatment using ruxolitinib leads to a reduction in CD3+T cells and decreased cytokine production. Besides regulatory T cells and Th1 cells [6–8] also effector functions of CD8+T cells are inhibited, which may be highly relevant in the context of SARS-CoV-2 induced immune responses. Even though the spectrum of JAK inhibition is broad, the suppression of cellular functions is cell type specific and incomplete. Therefore JAK inhibition was well tolerated by MPN patients in randomized clinical trials [9, 10] with a relatively low rate of infectious complications [11]. Accordingly, ruxolitinib should be considered an immunomodulatory drug rather than a classical immunosuppressive treatment. Besides its high efficacy in MPN, ruxolitinib has shown tremendous benefit in the treatment of acute and chronic GvHD after allogenic stem cell transplantation. Elegant preclinical studies and consecutive clinical trials investigating ruxolitinib in steroid-refractory GvHD [12, 13] have shown limited proinflammatory cytokine production as well as Th1 and Th17 polarization resulting in significantly improved survival. Likewise, in vitro and in vivo models of HLH revealed IFNγ dependent and independent mechanisms of ruxolitinib [14], which translated into improvement of cytopenia, transfusion independence, discontinuation of immunosuppressive treatment, and early hospital discharge in early clinical trials [15].

Here, La Rosée et al. have compiled an interesting series of patients severely affected from SARS-CoV-2 induced hyperinflammation and treated with ruxolitinib [16]. Out of 105 patients that were admitted for CoVID-19 infection, 14 critically ill individuals were identified by using a newly developed CoVID-19 Inflammation Score (CIS) and therefore qualified for ruxolitinib treatment. Ruxolitinib was administered for a median of 9 days and with a median cumulative dose of 135 mg, which resulted in efficient reduction of hyperinflammation as measured by clinical improvement (CIS score reduction), reduction of inflammation parameters (ferritin, CRP, IL6, and sIL2R), and the patients’ recovery rate. Of note, 85.7% of patients showed significant reduction of the CIS score, with sustained clinical improvement in 78.6% of cases. Only 1/14 ruxolitinib treated individuals did not survive, which is notable and unexpectedly low for investigating a high-risk subset of CoVID-19 patients. This report is especially encouraging in the light that none of the patients experienced significant toxicity and treatment duration was rather short. In an exemplary manner, the retrospective analysis presented here has already been translated in the initiation of a prospective multicenter clinical phase 2 trial (NCT04338958) that aims to confirm these findings and to assess for efficacy and safety in a controlled setting.

Several aspects of this report are of utmost interest: the choice of ruxolitinib needs to be weighed against other JAK- or cytokine inhibitors currently studied in clinical trials for CoVID-19 [17]. Importantly, immunosuppressive effects of JAK inhibitors are cell type dependent, determined by their specificity [18] and show a high variability in regard to their toxicity profile. Other JAK inhibitors such as tofacitinib are known to compromise B-cell differentiation and antibody production [19], which may be limiting in the light of reports highlighting the severity of CoVID-19 disease in B cell depleted patients and the anticipated need for antibody production to clear the virus. Use of others, such as baricitinib, may be limited by their risk profile regarding thromboembolic complications. Thromboembolic complications are highly relevant in critically ill CoVID-19 patients, where coagulation abnormalities are frequently reported and correlate with dismal prognosis. In contrast, the use of ruxolitinib has shown protective effects regarding thromboembolic events in preclinical studies [20] and advanced clinical trials [21]. Inhibitors of specific cytokine signaling pathways such as the IL6 receptor antibody tocolizumab have also been studied in CoVID-19 with variable success. As IL6 signaling has been identified as a major mediator of the SARS-CoV-2 induced immune response, tocolizumab has been described as specifically beneficial for patients with massively elevated IL6 serum levels. However, considering the ability of ruxolitinib to inhibit a wide range of proinflammatory cytokines by blocking signal transduction at the receptor level, it is tempting to speculate that particularly patients with the highest IL6 serum concentrations may benefit the most from JAK-inhibitor treatment.

Several questions remain unanswered: Which patients are the ones at risk for developing the hyperinflammatory syndrome? Those with high expression of angiotensin-converting enzyme (ACE) receptors in their lungs making them particularly vulnerable to CoVID-19 infections? While density of ACE receptors could explain a higher rate of virus entry, it does not necessarily explain the differences observed regarding immune responses and cytokine production. Are hematopoietic stem and progenitor cells hampered by activation of the Nlrp3 inflammasome, which is central to several pathological signaling pathways during hyperinflammation [22]? Most recently, clonal aberrations detected in the hematopoietic system have been reported to predispose otherwise healthy individuals to increased inflammation in the cardiovascular system and cardiovascular disease. Although highly speculative, the higher rate of clonal aberrations (especially JAK2 or TET2 mutations) in the hematopoietic system of older patients, could potentially enhance the SARS-CoV-2 induced hyperinflammation.

One last aspect, however, is not questionable: with mankind eagerly waiting for effective and safe medication to treat CoVID-19, drugs should not be tried with trial and error, as patients deserve appropriate care and academic assessment of novel therapies. With several promising compounds at hand, cherry picking of random candidate drugs is not appropriate and prevents recruitment of patients for clinical trials that are urgently needed to answer open questions. As exemplified by La Rosée and colleagues, positive data obtained in relevant case series should be rapidly translated into structured clinical trials. Withholding patients from such clinical trials due to formalities, conflicting interests or lack of personal experience during a deadly pandemic is neither ethical nor academically sound. These critically ill CoVID-19 patients do indeed have ‘something to lose.’
